# Campbell title registrations to date – May 2024, and discontinued protocols

**DOI:** 10.1002/cl2.1418

**Published:** 2024-06-16

Details of new titles for systematic reviews or evidence and gap maps that have been accepted by the Editor of a Campbell Coordinating Group are published in each issue of the journal. If you would like to receive a copy of the approved title registration form, please *send an email* to the Managing Editor of the relevant Coordinating Group.

A list of discontinued protocols appears below these new titles. If you are interested to continue a project, please get in touch with the Managing Editor of the relevant Coordinating Group or email info@campbellcollaboration.org.

## AGEING

Psychological antecedents of financial planning for retirement in working‐age adults: A scoping review

Zafira Shabrina, Olaf Simonse, Mirre Stallen, Lotte van Dillen, Wilco van Dijk

21 May 2024

Attitudinal factors related to the use of digital technologies in health by older adults: An overview of reviews

Elzbieta Malgorzata, Bobrowicz Campos, Cristina Camilo, Guilherme Pinheiro

4 May 2024

## CHILDREN AND YOUNG PERSONS WELLBEING

Association of antenatal cytokine concentrations with neurodevelopmental disorders of the offspring: A scoping review

Imasha Jayasinghe, Hannah Jones, Scott Graham, Kyle Eggleton, Russell Dale, Jane Alsweiler

3 April 2024

Improving information understanding by the child‐patients and the parents: A review of interventions

Julie Lienard, Gaelle Guyon, Vivien Garcia, Frédérique Claudot

3 April 2024

Effect of regulated recreational cannabis marketing policies on the consumption of adolescents from socially disadvantaged groups: A systematic review of the literature

Agustín Mora‐Concha, Francisca Roman Mella, Sebastián Neira

11 March 2024

## INTERNATIONAL DEVELOPMENT

Gender‐responsive macro‐level policies and women's economic empowerment in sub‐Saharan Africa: An evidence and gap map

David Sarfo Ameyaw, Takyiwaa Manuh, Sheila Agyemang Oppong, Clarice Panyin Nyan

14 February 2024

Effects of marketing strategies on upcycled food acceptance: A systematic review and meta‐analysis

Shuai Ma, Zhihong Xu, Peng Lu, Jean Parrella, Ashlynn Kogut

22 February 2024

Does intermittent fasting combined with calorie restriction versus calorie restriction alone provide additional health benefits when calories are equated?

Mohammed Hamsho, Wijdan Shkorfu, Yazan Ranneh, Abdulmannan Fadel

8 May 2024

## SOCIAL WELFARE

Resolving jingle‐jangle fallacies in self‐compassion research

Ramona Schöne‐Hoffmann, Manuela Benick, Dorota Reis, Sarah Schäfer, Franziska Perels

10 March 2024

The effect of digital intervention programmes for improving parent‐adolescent communication on sexual health and relationship: A systematic review

Laura Joseph, Pascal Navelle, Chinwendu Ngozi, Abdulmajeed Abdulsalam, Chimdindu Okafor, Rebekah McNaughton, Lawrence Nnyanzi

2 April 2024

Proactive resilience programmes for improving resilience and psychological adaptation in employees in high‐risk occupations: A systematic review

Trine Filges, Geir Smedslund, Anja Bondebjerg, Elizabeth Bengtsen

29 April 2024

The effect of adverse childhood experiences on employment outcomes: Protocol for a systematic review

Amarech Obse, Paul McCrone, Evdoxia Gkaintatzi

29 February 2024

PrEP (non)adherence among men who have sex with men: An overview of reviews

Guilherme Pinheiro, Carla Moleiro, David Rodrigues

23 April 2024

Complementarity between informal care and formal care to adults: Knowledge mapping through a scoping review of the literature

Marta Matos, Elzbieta Malgorzata Bobrowicz Campos, Rosa Silva, Francisca Castanheira, Diana Santos

29 February 2024

Exploring self care in the context of addictions: A scoping review protocol

Aina Folgueiras Vila, Maria‐Antonia Martorell‐Poveda, Maria‐del‐Señor Sesmilo‐Martínez, Pilar Vidal‐Massot, Laura Ortega‐Sanz

12 May 2024

## DISCONTINUED PROTOCOLS

If you are interested to continue one of the projects below, please get in touch with the Managing Editor of the relevant Coordinating Group or email info@campbellcollaboration.org.

## CRIME AND JUSTICE

https://onlinelibrary.wiley.com/doi/10.1002/CL2.85

PROTOCOL: Mandatory arrest for misdemeanor domestic violence effects on repeat offending

Barak Ariel, Lawrence W. Sherman

## EDUCATION

https://onlinelibrary.wiley.com/doi/10.1002/CL2.163

PROTOCOL: Provision of information and communications technology (ICT) for improving academic achievement and school engagement in students aged 4–18

Kristin Liabo, Laurenz Langer, Antonia Simon, Kathy‐Ann Daniel‐Gittens, Alex Elwick, Janice Tripney

https://onlinelibrary.wiley.com/doi/10.1002/CL2.210

PROTOCOL: Universal school‐based programmes for improving social and emotional outcomes in children aged 3–11 years

Paul Connolly, Sarah Miller, Jennifer Hanratty, Jennifer Roberts, Seaneen Sloan

https://onlinelibrary.wiley.com/doi/10.1002/CL2.110

PROTOCOL: Education support services for improving school engagement and academic performance of children and adolescents with a chronic health condition

Michelle A. Tollit, Susan M. Sawyer, Savithiri Ratnapalan, Tony Barnett

https://onlinelibrary.wiley.com/doi/10.1002/CL2.197

PROTOCOL: Electronic mentoring to promote positive youth outcomes for young people under 25: A systematic review

Robyn M. O'Connor, David L. DuBois, Lucy Bowes

https://onlinelibrary.wiley.com/doi/10.1002/CL2.166

PROTOCOL: Merit pay programs for improving teacher retention, teacher satisfaction, and student achievement in primary and secondary education: A systematic review

Gary Ritter, Julie Trivitt, Leesa Foreman, Corey DeAngelis, George Denny

## INTERNATIONAL DEVELOPMENT

https://onlinelibrary.wiley.com/doi/10.1002/cl2.1122

PROTOCOL: Development evaluations in India 2000–2018: A country impact evaluation map

Ashrita Saran, Eti Rajwar, Bhumika T. V., Divya S. Patil, Howard White

https://onlinelibrary.wiley.com/doi/10.1002/cl2.1186

PROTOCOL: The association between whole‐grain dietary intake and noncommunicable diseases: A systematic review and meta‐analysis

Wasim A. Iqbal, Gavin B. Stewart, Abigail Smith, Linda Errington, Chris J. Seal

https://onlinelibrary.wiley.com/doi/10.1002/CL2.84

PROTOCOL: Nutrition interventions and programs for reducing mortality and morbidity in pregnant and lactating women and women of reproductive age: A systematic review

Philippa Middl

https://onlinelibrary.wiley.com/doi/10.1002/CL2.171

PROTOCOL: Agronomic biofortification strategies to increase grain zinc concentrations for improved nutritional quality of wheat, maize and rice: A systematic review

Israel F. N. Domingos, Marcin Baranski, Carlo Leifert, Ismail Cakmak, Zed Rengel, Paul E. Bilsborrow, Gavin B. Stewart

https://onlinelibrary.wiley.com/doi/10.1002/CL2.138

PROTOCOL: Improving maternal, newborn and women's reproductive health in crisis settings

Primus Che Chi, Henrik Urdal, Odidika U. J. Umeora, Johanne Sundby, Paul Spiegel, Declan Devane

https://onlinelibrary.wiley.com/doi/10.1002/CL2.183

PROTOCOL: The impacts of interventions for female economic empowerment at the community level on human development

Marcela Ibanez, Sarah Khan, Anna Minasyan, Soham Sahoo, Pooja Balasubramanian

https://onlinelibrary.wiley.com/doi/10.1002/CL2.154

PROTOCOL: Peer‐based interventions for reducing morbidity and mortality in HIV‐positive women

J. Petkovic, M. Doull, A. M. O'Connor, J. Aweya, M. Yoganathan, V. Welch, G. A. Wells, P. Tugwell

https://onlinelibrary.wiley.com/doi/10.1002/CL2.188

PROTOCOL: Community‐led total sanitation in rural areas of low‐ and middle‐income countries: A systematic review of evidence on effects and influencing factors

Josef Novotný, Jiří Hasman, Martin Lepič, Vít Bořil

https://onlinelibrary.wiley.com/doi/10.1002/CL2.140

PROTOCOL: Access to electricity for improving health, education and welfare in low‐ and middle‐income countries

Kavita Mathur, Sandy Oliver, Janice Tripney

https://onlinelibrary.wiley.com/doi/10.1002/CL2.157

PROTOCOL: Saving promotion interventions for improving saving behaviour and reducing poverty in low‐ and middle‐income countries

Janina Isabel Steinert, Ani Movsisyan, Juliane Zenker, Ute Filipiak, Yulia Shenderovich

https://onlinelibrary.wiley.com/doi/10.1002/CL2.172

PROTOCOL: The effect of interventions for women's empowerment on children's health and education

Sebastian Vollmer, Sarah Khan, Le Thi Ngoc Tu, Atika Pasha, Soham Sahoo

https://onlinelibrary.wiley.com/doi/10.1002/CL2.200

PROTOCOL: The effects of road infrastructure, and transport and logistics services interventions on women's participation in informal and formal labour markets in low‐ and middle‐income countries

Manisha Gupta, Souvik Bandyopadhyay, Meerambika Mahapatro, Shreya Jha

https://onlinelibrary.wiley.com/doi/10.1002/cl2.1135

PROTOCOL: Water, sanitation and hygiene for reducing childhood mortality in low‐ and middle‐income countries

Hugh Sharma Waddington, Sandy Cairncross

## SOCIAL WELFARE

https://onlinelibrary.wiley.com/doi/10.1002/cl2.1259

PROTOCOL: Effects of virtual reality exposure therapy versus in vivo exposure in treating social anxiety disorder in adults: A systematic review and meta‐analysis

Martin Polak, Norbert Tanzer, Per Carlbring

https://onlinelibrary.wiley.com/doi/10.1002/CL2.109

PROTOCOL: The therapeutic alliance and psychotherapy outcomes for young adults aged 18 to 34

Stacy J. Green, Julia H. Littell, Karianne Hammerstrøm, Emily Tanner‐Smith, Jessica Schaffner Wilen

https://onlinelibrary.wiley.com/doi/10.1002/CL2.56

PROTOCOL: Cognitive‐behavioural therapy for parents who have physically abused their children

Mogens N. Christoffersen, Jacqueline Corcoran, Diane DePanfilis, Claire Daining

